# Editorial: Association between oral microbiota dysbiosis and the development of systemic conditions

**DOI:** 10.3389/fcimb.2023.1204103

**Published:** 2023-04-18

**Authors:** Zheng Zhang, Jian Zhou, Dong Xia, Zuomin Wang

**Affiliations:** ^1^ Tianjin Stomatological Hospital, School of Medicine, Nankai University, Tianjin, China; ^2^ Tianjin Key Laboratory of Oral and Maxillofacial Function Reconstruction, Tianjin, China; ^3^ The School and Hospital of Stomatology, Capital Medical University, Beijing, China; ^4^ Royal Veterinary College, University of London, London, United Kingdom; ^5^ Department of Stomatology, Beijing Chao-Yang Hospital, Capital Medical University, Beijing, China

**Keywords:** oral microbiota, dysbiosis, systemic diseases, inflammatory, mechanisms

Human oral cavity harbors a complex ecosystem of numerous microorganisms, including bacteria, fungi, viruses, and bacteriophages, referred to as the oral microbiota(Peng et al., 2022). In a healthy state, the oral microbiota is composed of commensals. The mutual commensal oral microbiota plays a crucial role in promoting not only oral, but also systemic health (Han et al., 2022). However, in a diseased state, host-microbial networks lead to dysbiosis. The influence of oral microbiota dysbiosis can extend beyond the oral cavity, and some systemic conditions are also associated with the oral microbiota (Peng et al., 2022). A mechanistic understanding for such associations may arise from the ability of many oral microorganisms to alter the inflammatory microenvironment and to interfere with host signaling pathways that control cell viability, proliferation and differentiation.

Furthermore, there is likely to be an alteration in oral microbial composition or bacterial pathogenicity in systemic pathologic conditions (Graves et al., 2019). Therefore, there may be a bidirectional relationship between the oral microbiota and systemic diseases. The abundance of oral microbiota specifically associated with systemic diseases appears to be a clinical biomarker to some extent, although further exploration is still necessary. If this clinical phenomenon is confirmed, early prediction of systemic diseases using microbial detection might be a crucial breakthrough. In the present research topic, we aim to assemble original research articles and reviews discussing recent advancements in understanding the association between oral microbiota dysbiosis and the development of systemic conditions.

## Alteration in oral microbial composition in systemic pathologic conditions

Oral microbiomes are closely related to the health status of the body. Oral squamous cell carcinoma (OSCC), one of the most common malignant tumors of the head and neck, is closely associated with the dysbiosis of the oral microbiome. Nie et al. analyzed microbial samples collected from six oral niches, including OSCC tumor tissue, adjacent normal tissue, cancer surface tissue, anatomically matched contralateral normal mucosa, saliva, and tongue coat. They found significant differences in microbial composition and function among the six different oral niches, and most of the genera significantly enriched in tumor tissue samples were those that have been associated with periodontal diseases. Nasopharyngeal carcinoma (NPC) is another common head and neck cancer. Hao et al. established a non-invasive and low-cost NPC risk screening model based on the oral and nasopharyngeal microbiome. The oral microbiome accuracy rate for NPC risk screening was reported to reach 77.2%.

Significant increases in circulating levels of estrogen and progesterone are thought to have a significant effect on the periodontium throughout pregnancy (Wu et al., 2015). The study by Zhang et al. compared the microbial community characteristics of supragingival plaque in 30 pregnant and 10 non-pregnant women, and indicated a trend toward increased oral microbial diversity during pregnancy. The imbalance of local and systemic immunity and metabolism in HIV-infected/AIDS patients can cause an imbalance in oral ecology (Tappuni and Sufiawati, 2020). Cao et al. demonstrated that highly active antiretroviral therapy (HAART), the most effective therapy for HIV infection, has a key role in the balance of salivary microecology in AIDS patients. However, there are still some systemic diseases that do not result in significant changes in oral microbiomes. Lu et al. explored the impact of diabetes on periodontitis-related microorganisms in the saliva, and showed that diabetes did not affect the diversity of salivary microbiota in the context of periodontitis.

## Effects of oral microorganisms on systemic diseases

The detrimental effects of oral microorganisms are not confined to the oral cavity, they can also contribute to systemic disorders, such as type 2 diabetes mellitus, cardiovascular disease, and inflammatory bowel disease (IBD) (Hajishengallis and Chavakis, 2021). The study by Kang et al. evaluated the influence of periodontal organism on glucose metabolism disorder in mice. After 22 weeks of periodontal pathogen infection, significant differences were observed at 30 and 60 min for the glucose tolerance test (GTT) and at 15 min for the insulin tolerance test (ITT). In the review by Li et al. the authors provided a comprehensive overview on the influence of periodontitis and the effect of periodontal therapy on vascular endothelial function, and the molecular mechanisms involved. In the article by Chen et al. the influence of periodontal pathogen infection on gut mycobiome was explored. The authors demonstrated the first evidence of gut fungal dysbiosis with periodontal pathogen administration. Wang et al. further reported that periodontitis salivary microbiota gavage could exacerbate intestinal inflammation, and aggravate long bone loss through the oral-gut axis in ovariectomized (OVX) rats.

## Pathogenic mechanism of oral microorganisms

The presence of integrin β6 (ITGB6), an epithelial-specific receptor, is essential for periodontal health, as its low expression in the gingival epithelium is related to the development of periodontal diseases (Ghannad et al., 2008). An interesting research paper contributed by Xu et al. showed that the interaction of the transcription factors forkhead box protein O1 (FOXO1) and signal transducer and activator of transcription 3 (STAT3) mediated oral bacterial biofilm components induced ITGB6 downregulation in human epithelial cells, and speculated that targeting the FOXO1–STAT3 signaling axis might be an effective method for treating periodontal diseases. Previous research has confirmed that potassium ion is associated with enhanced virulence of the plaque community *in vitro*(Yost et al., 2017). The study by Zou et al. deciphered the role of TrkA, the regulatory subunit of the potassium uptake system Trk in periodontal pathogen-*Porphyromonas gingivalis* (*P.gingivalis*). The authors found that TrkA is required for the virulence of *P.gingivalis in vivo*.

It has been concluded that *P.gingivalis* or its virulence factors can induce memory impairment and Alzheimer’s disease (AD)-related pathologies (Jiang et al., 2021; Tang et al., 2021). Outer membrane vesicles (OMVs), secreted by Gram-negative bacteria, could carry bacterial LPS, protease, membrane receptor, DNA, RNA, and so forth, and act as long-distance weapons and cause systemic disease (Toyofuku et al., 2019). In the study by Gong et al. the authors demonstrated the relationship between *P.gingivalis* OMVs and AD-like phenotypes for the first time. This study indicated that oral gavage of *P.gingivalis* OMVs could induce AD-like phenotypes, including memory dysfunction, neuroinflammation, and tau phosphorylation, and NLRP3 inflammasome activation was a possible mechanism.

## Conclusion

In summary, the articles presented in this research topic provide readers with updated data on the association of oral microbiota dysbiosis with systemic conditions. We hope that the knowledge from these published articles will be useful for initiating new studies in this exciting area where the detailed or acquired molecular mechanisms remain unexplored.

## Author contributions

All authors listed have made a substantial, direct and intellectual contribution to the work, and approved it for publication.
